# Supplementary material for: Spatial immunization to abate disease spreading in transportation hubs
Source: Nat Commun. 2023 Mar 20;14:1448. doi: 10.1038/s41467-023-36985-0 (PMC10027826; doi:10.1038/s41467-023-36985-0)
Supplement: Supplementary file 1 — Supplementary Information [file 41467_2023_36985_MOESM1_ESM.pdf]

Supplementary Information
Spatial immunization to abate disease spreading in transportation hubs

Mattia Mazzoli,* Riccardo Gallotti, Filippo Privitera, Pere Colet, and José J. Ramasco†

* mattia.mfn@gmail.com
† jramasco@ifisc.uib-csic.es

Data treatment 2
Supersampling . . . 2
Trajectories reconstruction . . . 2
Scaling properties of the reconstruction . . . 2
Travelers classification . . . 3
Modeling 4
Modeling epidemics in open systems . . . 4
Contact patterns heterogeneities . . . 5
The interplay of contacts and infections on hotspots designation. . . . 8
Temporal scale effect . . . 10
Applicability 11
Data representativeness . . . 11
Generalizability of the method . . . 11
References 15

DATA TREATMENT

Supersampling

In order to reach a realistic amount of users per day we have to upscale our data, by considering that all the trajectories recorded take place in the same day. To do this we consider all users with at least 10 points in their records and displace their trajectories in time to the same day. Every time we do this we generate a new random name for the time displaced user which depends on the day of appearance. This means that if one user appears in Heathrow on Monday and then again on Friday, it will appear on the standard day as being two different persons. When running simulations, we run the same standard day multiple times. Hence we assume travelers to get infected one day and come back clean on the next day as new users, in order to simulate a new flux of travelers. By the way we cannot do the same with workers. We need workers to get infected today and come back still infected tomorrow, without appearing as distinct workers.

By doing all this we pass to $223,000$, which is way beyond what we need. Although, we still have to filter our trajectories one more time with a procedure described next. See [1] for the supersampling code.

Trajectories reconstruction

We discretized space and time in order to have a temporal network approach, but still we lack some information which can be retrieved by few and simple hypotheses. Indeed one user could appear in one cell at a given time slot and then appear $40m$ away after one time slot. We lost the information on the trajectory along the path. We complete the missing data only inside the Terminals through the definition of an optimal path. Using the airport space grid parametrization we build a latter where cells are nodes and links define the first 4 neighborhood relations. We filter out those cells which have not been visited in the original data more than $10, 20, 30, 40$ and 50 times to avoid users appearing in unrealistic places. With this spatial network we build all the shortest paths for each couple of cells, except those who are more distant than $50m$, to avoid creating tortuous paths with no realistic meaning. On average we reconstruct around $4,000,000$ of new points in all the data, getting to $10,000,000$ points overall. For the sake of abbreviation we will refer to supersampled reconstructed trajectories data as SRTD10, SRTD20, SRTD30, SRTD40, SRTD50, depending on the thresholds used in the reconstruction process.

Scaling properties of the reconstruction

To be sure the process of supersampling and trajectories reconstruction is not producing any bias in our data, we compare the copresence matrix of the original data with the ones defined by the reconstructed data in three of the cases defined by the optimal path parameter (see Supplementary Figure S1a). As can be seen each element of the matrices scales with respect to the thresholds. Computing the rates of each element between the reconstructed data and the original ones, we can define a mean of these rates and plot Supplementary Figure S1a. This tells us that using a threshold of 20, 30 or 50 presences does not make any difference. Anyway, using weaker filters like 10 as the optimal path parameter, means to have way more encounters, maybe due to the use of non-representative places. We decide to use the value in the middle (30 presences) to avoid overestimating encounters. As a last verification, since

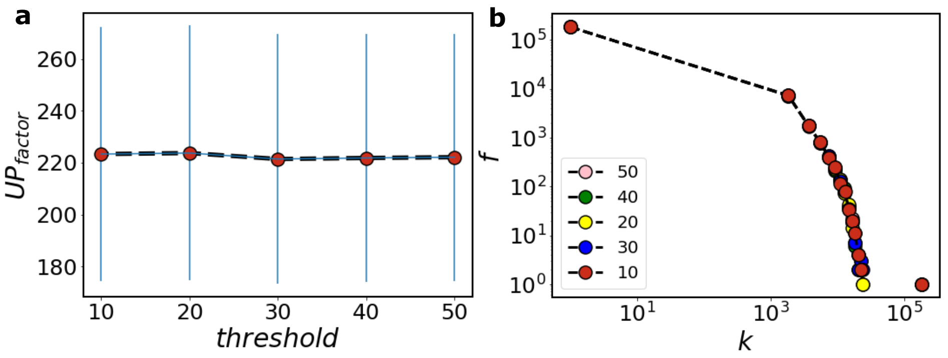

Supplementary Figure S1. **Reconstruction invariant properties.** **a** Mean upscaling factor as registered from the ratio between upscaled and original copresence matrix elements. Error bars represent the error of the mean, computed over the n = 16 copresence matrix elements. **b** Degree distributions of SRTD10, SRTD20, SRTD30, SRTD40, SRTD50 aggregated networks.

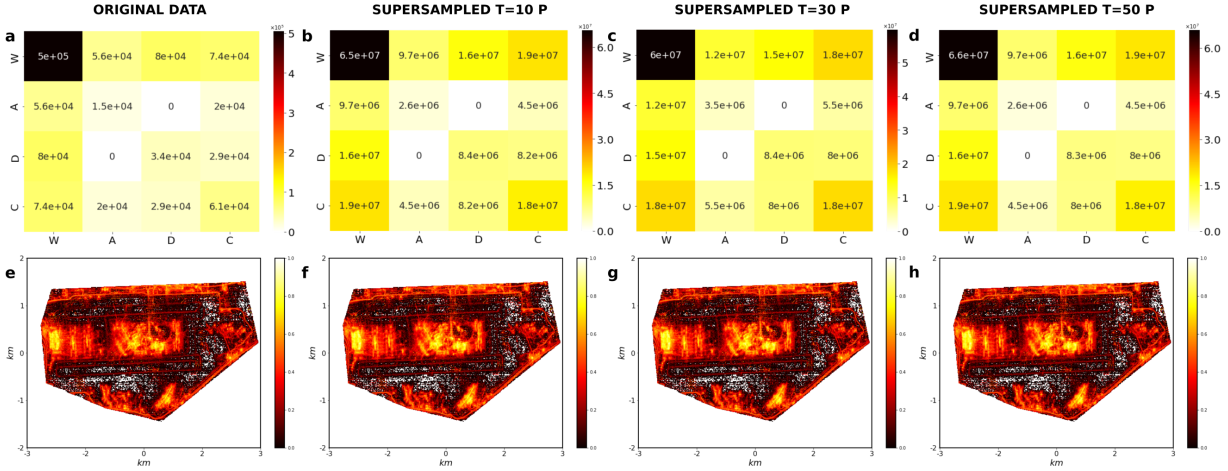

Supplementary Figure S2. **Reconstruction scaling properties.** **a,b,c,d** Original data, SRTD10, SRTD30 and SRTD50 copresence matrices. **d,e,f,g** Original data, SRTD10, SRTD30 and SRTD50 heatmaps of aggregated presences. Dark colors in panels a,b,c,d represent the highest number of contacts, light colors represent the lowest number of contacts. Yellow colors in panels e,f,g,h represent the densest areas, dark colors represent the least dense areas.

we are using a network approach, we build the aggregated copresence network for each kind of data and we draw the degree distribution. As we observe in Supplementary Figure S1b all of the reconstructed data show the same degree distribution curve, hence we can finally affirm our method is invariant with respect to the reconstruction parameters we choose.

Finally, in Supplementary Figure S2 we can also appreciate the scaling of the presences heatmaps. Each heatmap has been normalized in order to show all the detailed shades within the terminals. All the reconstructed data heatmaps show brighter plots inside corridors with respect to the one built on the original data. Presences in the landing strips are also brighter because of the initial supersampling adopted from the original data. Note that we do not apply any reconstruction on trajectories out of the terminals.

Travelers classification

By analyzing some specific places visited by users, see Supplementary Figure S3, we recognize among travelers those who are arriving, departing and connecting between flights, opening the door for further and specialized analyses on travelers behaviors. We detect further workers who appear in restricted areas like facilities or training programs buildings. Nonetheless, we also find trajectories of people who never appear in terminals nor facilities, for example car drivers appearing along the circuiting road. We discard these trajectories. To do this we go through a straight classification process.

For each user we register the first and the last point of their trajectory after the sumpersampling process and we

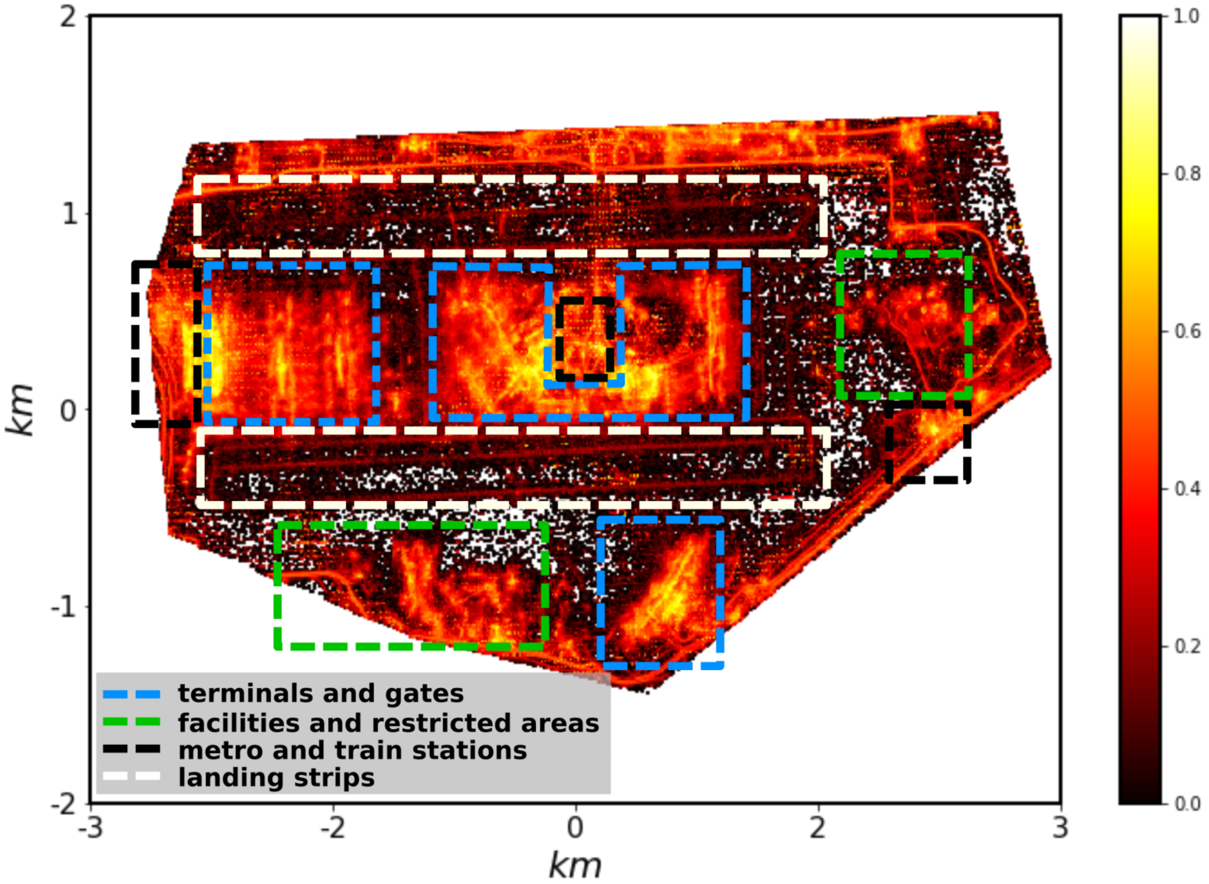

Supplementary Figure S3. **Trajectories classification areas.** Yellow colors represent the densest areas, dark colors represent the least dense areas.

check their positions with respect to terminal gates, metro stations, facilities and landing strips. Arrivals are those who appear at gates or landing strips and disappear somewhere else. People not appearing in any of the above mentioned places, but disappearing at metro stations, are also considered as arrivals. Connections are those users both appearing and disappearing at gates, no matter which ones. Departures are users appearing at metro stations but also those who disappear at gates or landing strips without appearing there at first. Further workers are those appearing and disappearing in facilities. People not appearing or disappearing in none of these places go through a further analysis, which is mentioned here. We label each one of their trajectories points with a letter and get a spatial semantic sequence for every trajectory: A for points in terminal 2 and 3 southern gates, B for terminal 2 gates, C for terminal 3 gates, D for terminal 4 gates, E for terminal 5 gates and P for landing strips. Last but not least, we recognize as workers those appearing at any moment at least once in facilities or restricted areas. If their sequences account for more than 3 diverse places, we consider them as workers moving to many terminals for job purposes, i.e. "A+D+E+B". We assume that travelers are visiting at most 3 places including the landing strips, e.g. "A+D+P". If their sequences account for the landing strip but not as the starting nor the ending point, i.e. "A+P+B", we label them as workers moving in the landing strips, e.g. landing operators, bus drivers, etc. Those who satisfy none of the above conditions, hence appear and disappear elsewhere or have sequences of only one character, are labeled as bad users and discarded. By this last filtering we finally get to $200,000$ users.

MODELING

Modeling epidemics in open systems

The optimal way to parametrize the model would be to have the estimation of R_0 and the contact rate from the literature for the population used in those studies. In this way, we could estimate p_β per contact and inform the

model with a contagion probability consistent with the original studies. R_0 is usually estimated with statistical models at population level, not considering individual level heterogeneities such as different individual contact rates. From a technical point of view, the contact rate observed in the airport for each individual is not representative of their contact rate in other settings. Using the contact rate measured in the airport without considering that this applies only for a fraction of their total infectious period would lead to underestimate the probability of infection p_β of the airport population.

Given that we need to use a certain value of p_β, we take two approaches: The first is to estimate p_β in order to recover the values of R_0 reported in the literature inside the airport facilities. This is done by informing the model with the contact rate β empirically measured in the airport to estimate the average transmissibility (or probability of infection per contact) p_β. Given an estimation of R_0 for a certain disease, we take into account two factors for the parameterization of p_β in the model:

- Airports are open systems, the population is never conserved in time, people arrive and leave changing the total susceptible population inside the system at all hours and, most importantly, reducing the real time of infectiousness of agents in the system. For a given R_0, infectious agents would be able to infect susceptibles only when they are present in the airport, reducing the number of secondary infections due to anticipated removal. To correct for this, we need to take into account the median fraction of time spent in the airport by all individuals (S4a), $f = 8\ time\ slots/96\ time\ slots = 2hours/24hours = 0.083$

- Airports are very crowded settings in which individuals produce many more contacts than the average general population and, hence, are not representative of an average individual's behavior. Moreover, these contacts are produced only in a fraction of the whole day, and hence, only during a fraction of the individual's infectious period. The number of unique contacts made in a time slot by each individual is highly heterogeneous along the day, hence we compute the average of the median contact rates at all time slots (blue solid line), $\beta = 34.6$ contacts/time slot, as shown in Fig. S4b. Since we do not have information on the contacts produced outside of the airport by the same individuals, we can correct this rate by the above measured fraction f of daytime spent in the airport. This leads to the adjusted contact rate $\beta' = 2.89$ contacts/time slot.

Finally $R_0 = p_\beta \beta'/\mu$, hence $p_\beta = R_0\mu/\beta'$ where μ is the probability of recovering expressed in 15minutes time slots rather than in days, as typically reported in the literature. See [1] for the example code of an SIR simulation.

This is a rough approximation to the real p_β of a given disease. Therefore, the second approach consists in a sensitivity analysis to make sure that our identification of the contagion hotspots and transmission reductions are robust. We performed further simulations for SARS-CoV2 exploring a range of different transmissibility values, with lower and higher values for p_β, recovering qualitatively similar results. In all the cases, the spatial immunization policy produced a sensible reduction of outbreak intensity, see Supplementary Figure S15.

Contact patterns heterogeneities

The distribution of contacts occurring per cell in a whole day and in each time slot shows high spatial heterogeneity. Figure S5 shows few cells with a high number of contacts and many cells with low amounts of contacts. Although the top ranked cells by number of contacts may not exactly correspond to the top ranked cells by number of contagions, from this distribution we see that most of the at-risk contacts are concentrated in a few cells in the airport. This explains the high efficacy of our method even with percentages of immunized cells lower than 2% (< 800 cells).

The distribution of inter-event times between activation times of a same link along the standard day encodes the burstiness of the temporal contact network, as shown in Fig. S6. We measured the burstiness parameter $B = -0.25$ following the methodology from [2].

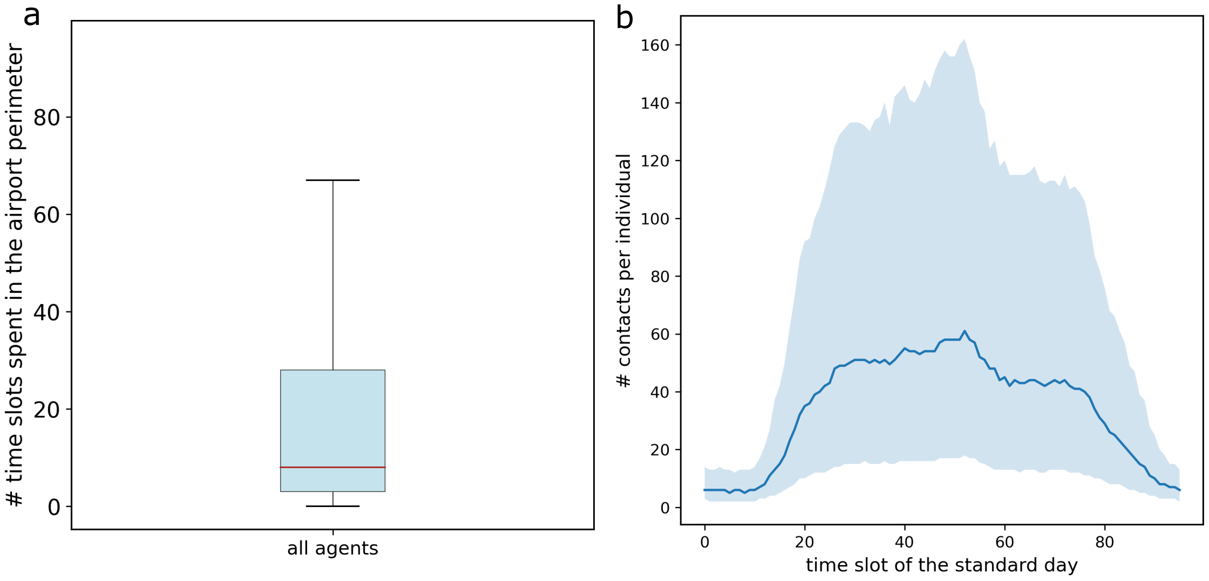

Supplementary Figure S4. **Model parameterization in an open system. a)** Time spent by the n = 206,043 agents in the airport perimeter during the standard day expressed in 15' time slots. Whiskers represent the $5-95\%$ interval, boxes the IQR, red line the median. Most agents spend less than 7 hours within the perimeter, but the distribution is highly skewed due to all type of workers, connecting and delayed passengers. **b)** Contact rate of agents along the standard day. Blue line represents the median of the distribution obtained from all agents' number of unique contacts at each time slot. Shaded area stands for the $5-95\%$ interval of the distribution obtained from all agents' number of unique contacts at each time slot.

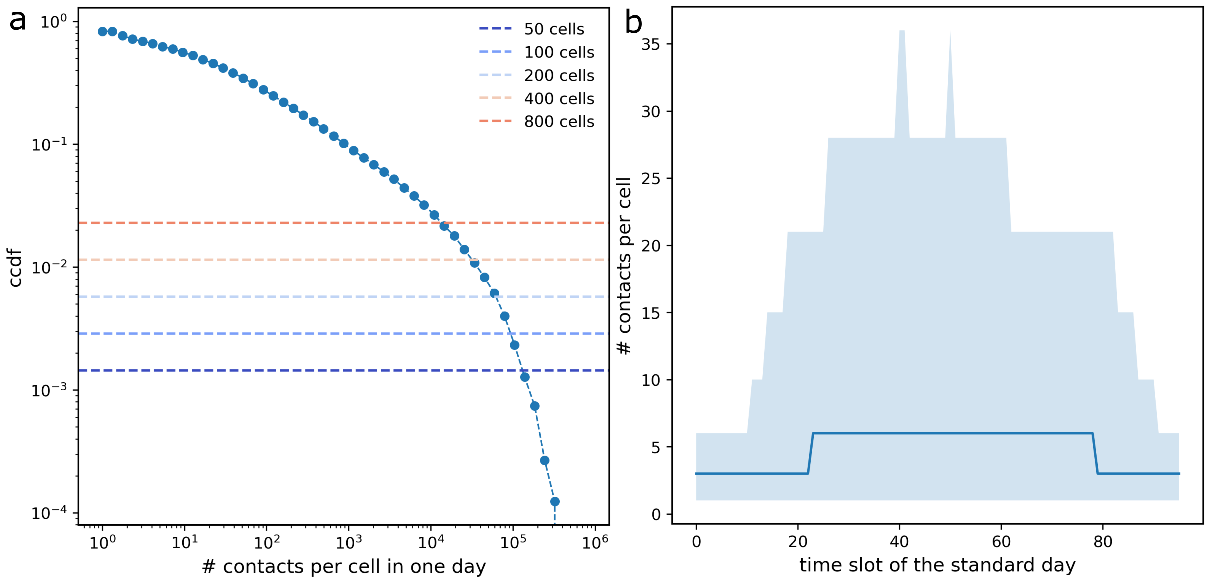

Supplementary Figure S5. **Number of unique contacts occurring per cell. a)** Distribution of the number of unique contacts occurring in each cell during the day. Dashed lines represent respectively the top 50, 100, 200, 400, 800 ranked cells by number of contacts in one day. **b)** Distribution of the number of unique contacts occurring in each cell at each time slot. Dashed area represents the $5-95\%$ interval, solid line represents the median.

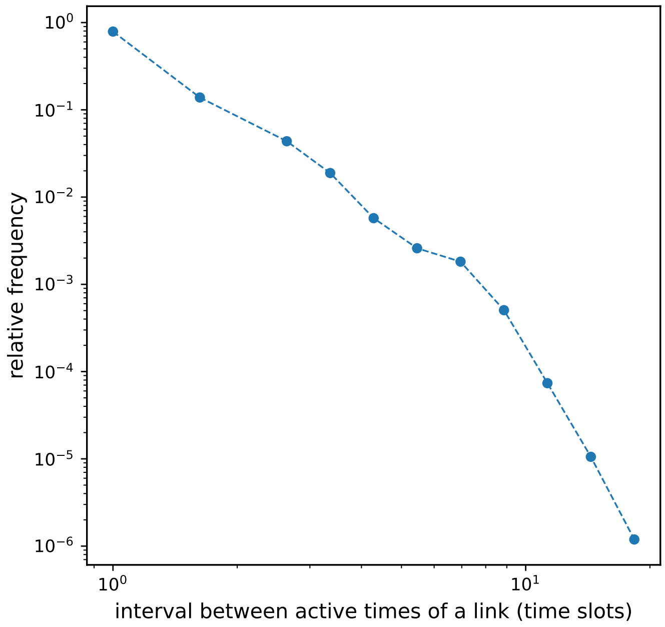

Supplementary Figure S6. **Burstiness of contacts.** Distribution of the interval between activation times of a same link along the standard day expressed in time slots of 15 minutes.

The interplay of contacts and infections on hotspots designation.

Most of the infectious hotspots observed in epidemic spreading simulations in the airport are often recurrent among other simulations with different infection parameters.

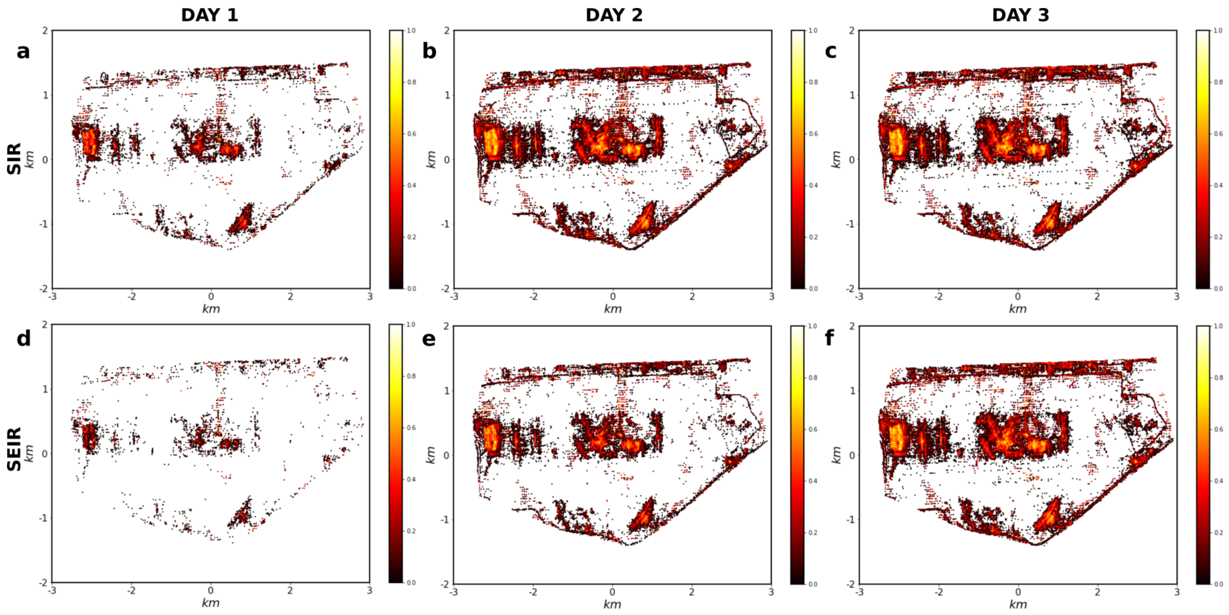

Supplementary Figure S7. **Contagions heatmap. a,b,c** Day 1,2 and 3 for SIR contagions. **d,e,f** Day 1,2 and 3 for SEIR contagions. Yellow colors represent the most infectious areas, dark colors represent the least infectious areas.

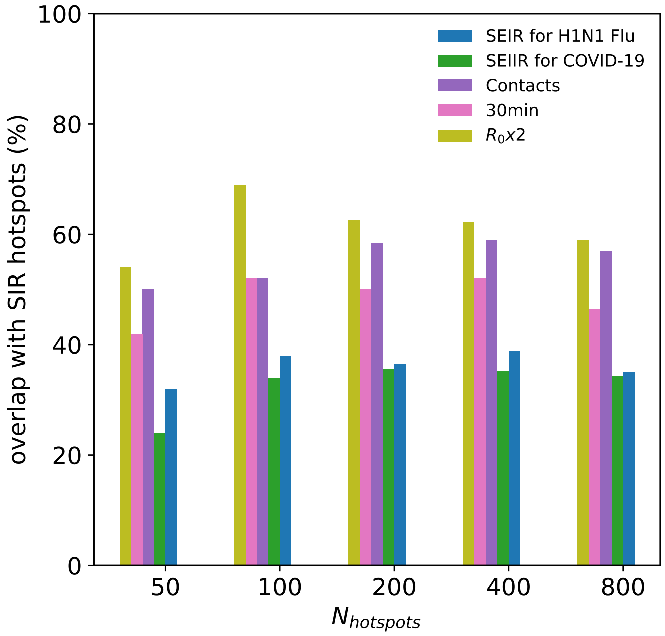

Supplementary Figure S8. **Hotspots overlap.** Percentage of hotspots overlap in different scenarios with respect to the SIR designed hotspots versus number of ranked hotspots considered.

Intuitively, using the SIR model, most of the infections occur in cells where most of the contacts happen, as shown in Supplementary Figure S9. Still, these most infectious cells do not strictly correspond to those where most of the contacts occur, indicating that the dynamics occurring in site are a little more complex than some kind of linearity

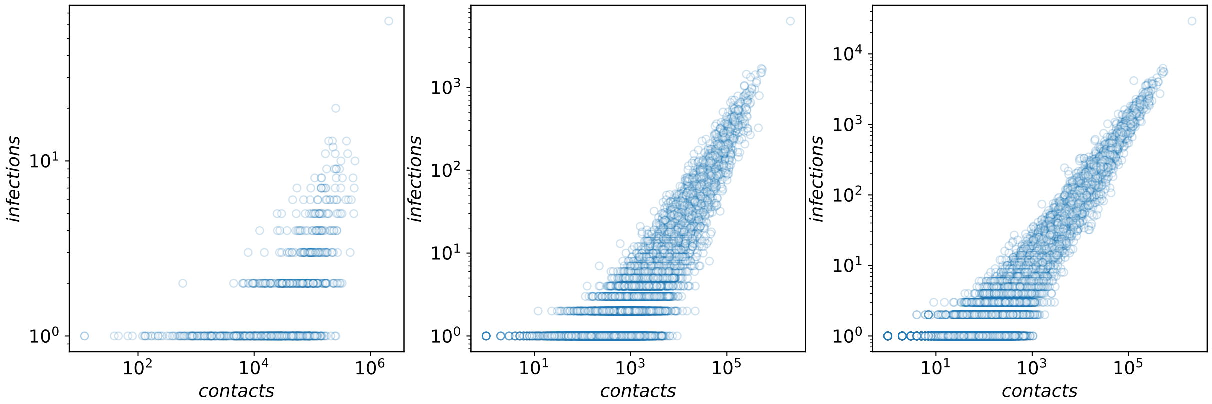

Supplementary Figure S9. **Infections vs contacts.** Cumulative infections versus contacts occurred in the same cells in $n = 1000$ SIR simulations on **a** the first day, **b** the second day, **c** the third day.

with the number of contacts.

Temporal scale effect

To check if the time scale of contacts and the infectiousness of the disease over time play any role in our results, we double the scale from 15 minutes to 30 minutes. We have to do this to check if the results are consistent.

This time scale switch yields a non-linear effect on the dynamics, since every cell stays infected the double of the time. An infected individual infecting a cell at the minute 0 of the simulation will cause a second individual passing by at minute 29 to get infected. This would have never happened under the assumption that the cell stays infected for 15 minutes only. Hence the second individual will get infected and the same space cell will stay infected for another 30 minutes, causing a chain reaction on all the other people passing by, who would have never got infected. As we see from Supplementary Figure S10, the number of infections occurring at every time step is more than double with respect to the 15 minutes case. In this case, the effect is super-linear given the prolonged infectiousness of cells.

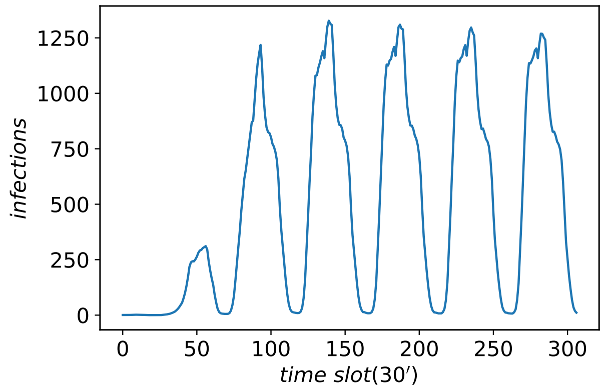

Supplementary Figure S10. **Infections frequency per time slot**. For each time slot of 30 minutes we count how many infections occurred.

Prolonging the infectiousness of cells over time is an exercise we have to do in order to understand what happens if viruses persist in the areas for a longer time.

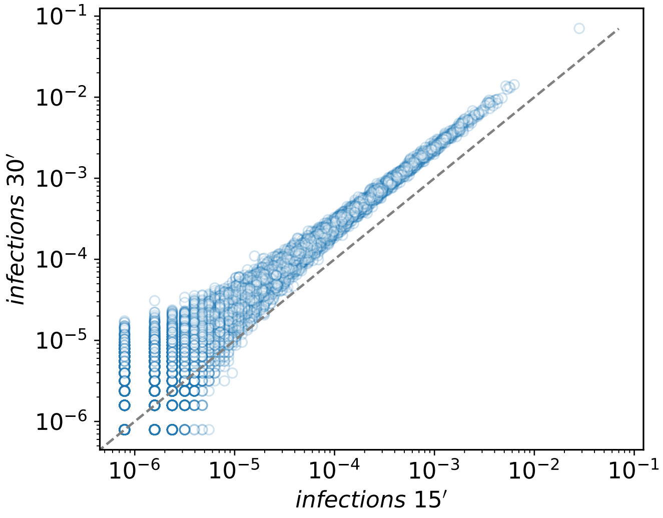

Supplementary Figure S11. **Cells infection probability** On the x-axis the rate of occurring infections in the first three days per each cell in the SIR $n = 1000$ simulations with time slots of 15 minutes, on the y-axis the same with time slots of 30 minutes. Dashed line represents identity.

We count what is the probability for an infection to occur in any space cell of the airport with $n = 1000$ simulations with the 15 minutes scale and the 30 minutes scale. The results confirm that the viral hotspots are not depending on

the time scale or on the cells infectiousness in time, as shown in Supplementary Figure S11.

APPLICABILITY

Data representativeness

In order to assess data representativeness of users' trajectories within the airport, we have to look into the data from the Civil Aviation Authority ([3], Table 11.7 for Heathrow airport). These data represent the stratification by age of passengers in Heathrow airport. People aged less than 19 years old represent approximately only the 5% of total passengers, while people aged 65+ represent approximately the 10% of total passengers. In general, most passengers are included in the range 25-54 years old. By looking at the percentage of smartphones usage in the UK stratified by age [4], we can see that the usage is pretty uniform in the spectrum ranging from 25-54 years old. From a mobility perspective, young children will always be accompanied by adults, i.e. their path will never be a completely different one with respect to the one of their parents/accompanists. From an epidemiological perspective, this results in strongly coupled trajectories between children and accompanists, hence strongly coupled disease statuses. By construction of our supersampling process, coupled trajectories are already present in our network. For all these reasons, we are confident that age bias is no concern for this type of study in these particular settings, i.e. transportation hubs.

The data may be underrepresenting passengers from other parts of the world. This may bring a slight bias to our analysis. However, we inferred destinations of infected agents by looking at the probability of flying from Heathrow terminals to countries of destinations from air traffic data. Air traffic records rely on official schedules of Heathrow airports hence shall not be affected by this kind of bias. Passengers flying to Cuebiq underrepresented regions of the world may be both residents of those regions or UK residents, and these latter users are not missing from the database. However, by looking at terminals' flights composition, we could not spot strong associations between airport terminals locations and traffic regarding specific world regions. Gates are not specifically dedicated to one unique region of the world. Hence, arrival passengers' nationalities will be generally mixed in all areas of the airport, therefore underweighting of contacts shall not affect specific areas of Heathrow airport. On the other hand, we have no information on incoming passengers regarding their flight origin from the mobility data, hence we cannot infer passenger's origins from their first appearance to correct for this potential misrepresentation. As a last remark, note that here we aggregated passengers' destinations into UK (domestic), European Union, London (incoming) and Intercontinental travelers. The latter includes the Americas, Asia, Africa and Oceania. Hence underrepresented areas of the world may once more be mixed to well-represented areas of the world. For this reason, we are confident that this kind of potential bias in the data shall not affect our results. For details on coverage bias in other parts of the world, see [5].

Generalizability of the method

We show results considering lower immunization efficacy, namely with 80% and 65% of p_β.

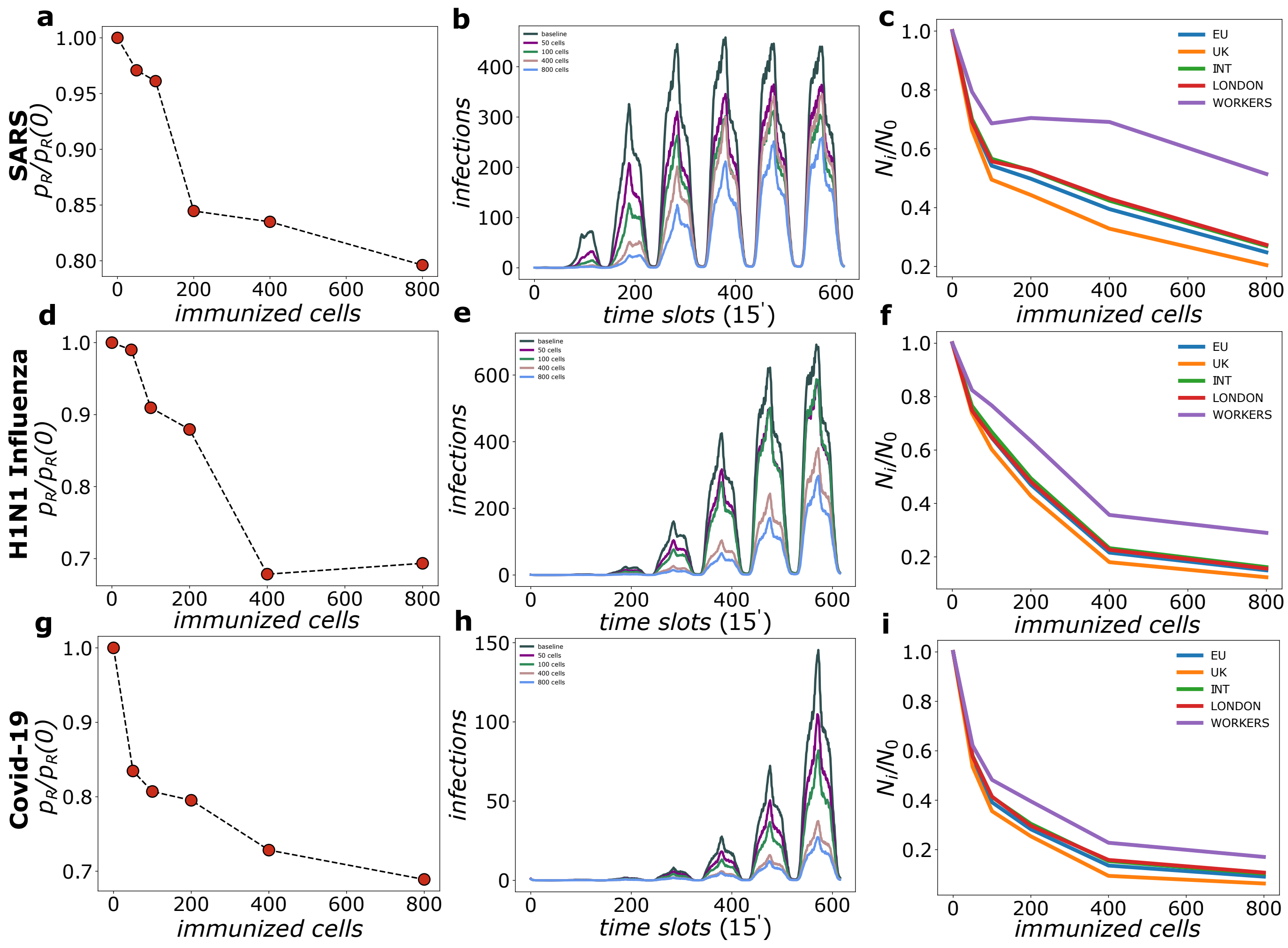

Supplementary Figure S12. **Spatial immunization applied to the spreading of diseases with efficacy of** 80%**.** Panels **a**, **b** and **c** refer to results of simulations for SARS; panels **d**, **e** and **f** refer to results of simulations for H1N1 Influenza; And panels **g**, **h** and **i** to those for COVID-19. In terms of columns, panels **a** and **d** show the number of realizations with secondary contagions p_R normalized by $p_R(0)$ (without spatial immunization) as function of the number of immunized cells. The cells to immunize are selected following the contagion ranking according to the SIR model. Panels **b**, **e** and **h** show the average number of new infections per time slot. Panels **c**, **f** and **i**, infections over the full simulation period disaggregated by workers and passenger destinations.

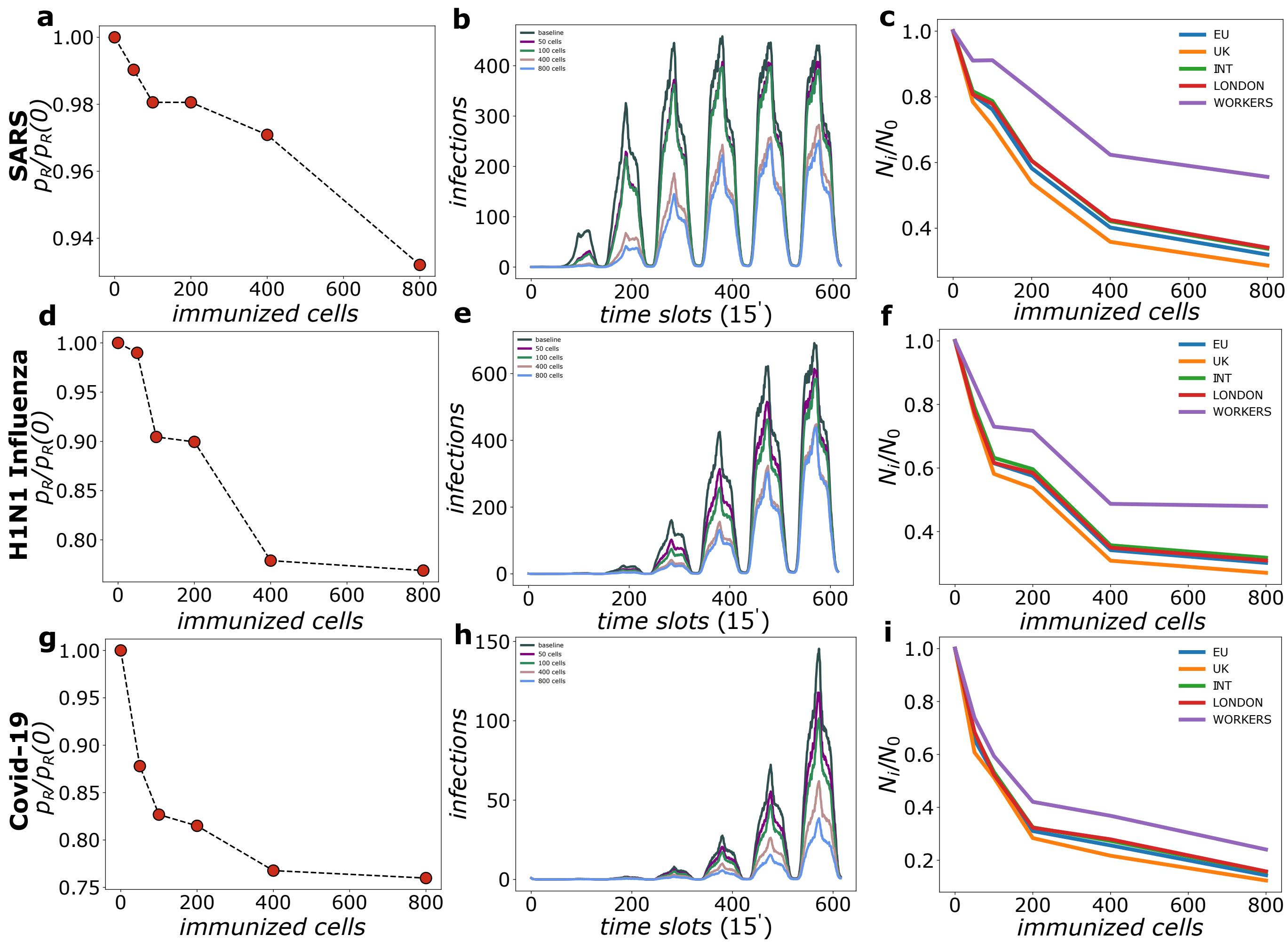

Supplementary Figure S13. **Spatial immunization applied to the spreading of diseases with efficacy of** 65%**.** Panels **a**, **b** and **c** refer to results of simulations for SARS; panels **d**, **e** and **f** refer to results of simulations for H1N1 Influenza; And panels **g**, **h** and **i** to those for COVID-19. In terms of columns, panels **a** and **d** show the number of realizations with secondary contagions p_R normalized by $p_R(0)$ (without spatial immunization) as function of the number of immunized cells. The cells to immunize are selected following the contagion ranking according to the SIR model. Panels **b**, **e** and **h** show the average number of new infections per time slot. Panels **c**, **f** and **i**, infections over the full simulation period disaggregated by workers and passenger destinations.

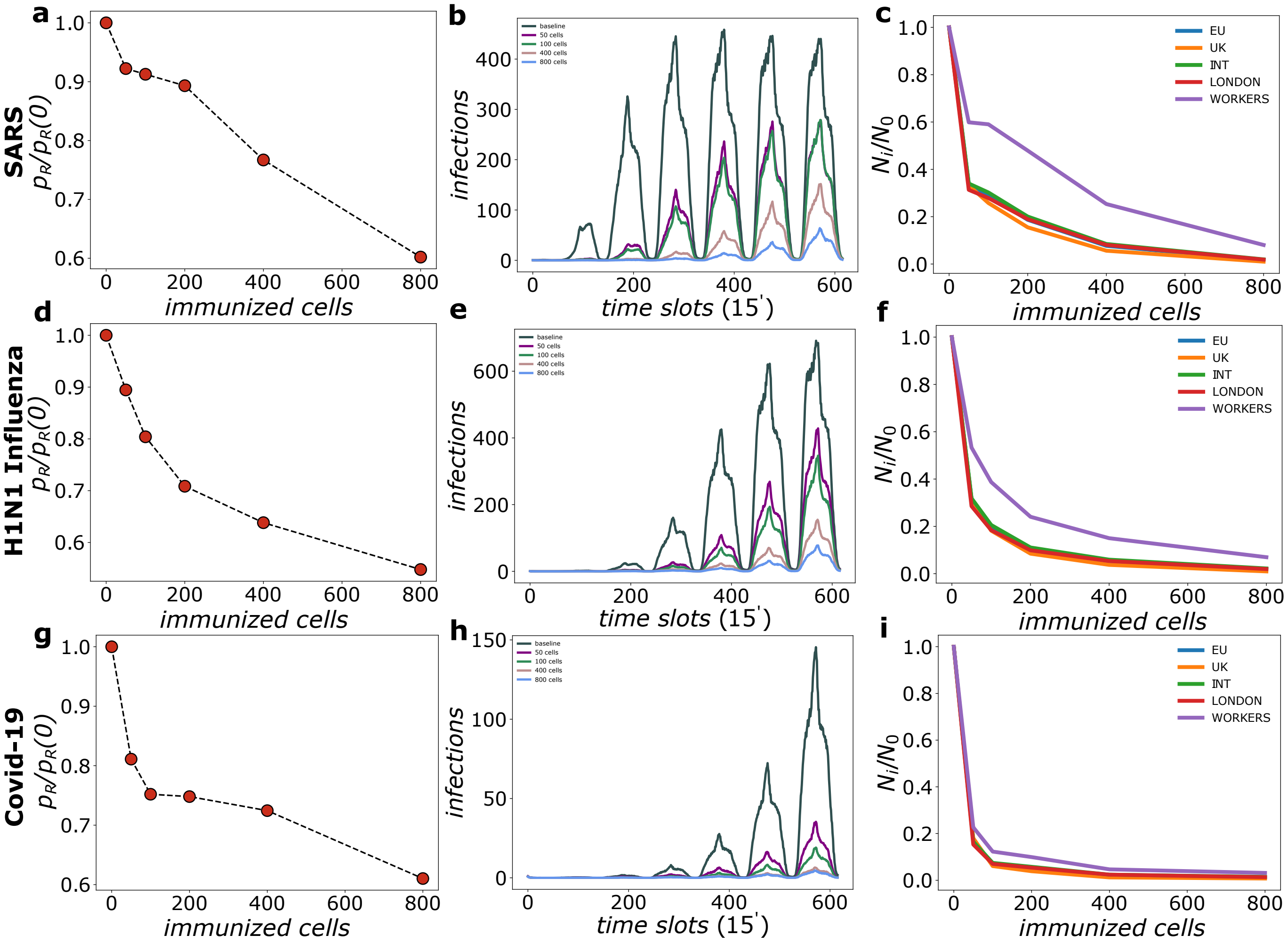

Supplementary Figure S14. **Spatial immunization applied to the spreading of diseases with slightly altered mobility.** Panels **a**, **b** and **c** refer to results of simulations for SARS; panels **d**, **e** and **f** refer to results of simulations for H1N1 Influenza; And panels **g**, **h** and **i** to those for COVID-19. In terms of columns, panels **a** and **d** show the number of realizations with secondary contagions p_R normalized by $p_R(0)$ (without spatial immunization) as function of the number of immunized cells. The cells to immunize are selected following the contagion ranking according to the SIR model. Panels **b**, **e** and **h** show the average number of new infections per time slot. Panels **c**, **f** and **i**, infections over the full simulation period disaggregated by workers and passenger destinations.

In order to check whether the SIR-designed hotspots suffer from overfitting to trajectories coming from our dataset, we applied a normal noise with mean $m = 0$ and standard deviation $\sigma = 15'$ to trajectories starting times.

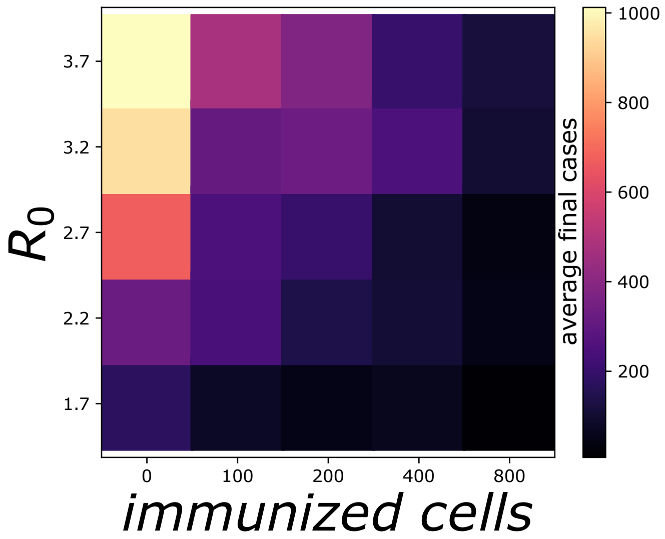

Supplementary Figure S15. **Policy efficacy with different values of R_0 for SARS-CoV2.** On the x-axis the R_0 of the virus, on the y-axis the number of irradiated space cells. Heatmap colored depending on the number of average final cases for each scenario after four days, light colors represent the highst number of total infections, dark colors represent the lowest number of total infections.

---

[1] Supersampling code and epidemic model example code in Figshare repository. `https://doi.org/10.6084/m9.figshare.19780192`. Accessed: 2023-01-05.

[2] Goh, K.-I. and Barabási, A.-L. Burstiness and memory in complex systems. *EPL (Europhysics Letters)* **81**, 48002 (2008).

[3] Civil aviation authority. `https://www.caa.co.uk/media/zs0lcx3t/t11_2018.pdf`. Accessed: 2022-12-19.

[4] Smartphone usage statistics in uk. `https://cybercrew.uk/blog/smartphone-usage-statistics-uk/`. Accessed: 2022-12-19.

[5] Let's talk about bias: A solution-oriented approach to representativeness in mobility data. `https://spectus.ai/social-impact/lets-talk-about-bias/`. Accessed: 2022-12-19.
