# Supplementary material for: Spatial immunization to abate disease spreading in transportation hubs
Source: Nat Commun. 2023 Mar 20;14:1448. doi: 10.1038/s41467-023-36985-0 (PMC10027826; doi:10.1038/s41467-023-36985-0)
Supplement: Supplementary file 3 — Reporting Summary [file 41467_2023_36985_MOESM3_ESM.pdf]

nature portfolio

Corresponding author(s): MATTIA MAZZOLI, JOSE JAVIER RAMASCO

Last updated by author(s): FEBRUARY 8 2023

Reporting Summary

Nature Portfolio wishes to improve the reproducibility of the work that we publish. This form provides structure for consistency and transparency in reporting. For further information on Nature Portfolio policies, see our Editorial Policies and the Editorial Policy Checklist.

Statistics

For all statistical analyses, confirm that the following items are present in the figure legend, table legend, main text, or Methods section.

n/a	Confirmed	
☐	☒	The exact sample size (*n*) for each experimental group/condition, given as a discrete number and unit of measurement
☐	☒	A statement on whether measurements were taken from distinct samples or whether the same sample was measured repeatedly
☒	☐	The statistical test(s) used AND whether they are one- or two-sided<br>*Only common tests should be described solely by name; describe more complex techniques in the Methods section.*
☒	☐	A description of all covariates tested
☒	☐	A description of any assumptions or corrections, such as tests of normality and adjustment for multiple comparisons
☐	☒	A full description of the statistical parameters including central tendency (e.g. means) or other basic estimates (e.g. regression coefficient) AND variation (e.g. standard deviation) or associated estimates of uncertainty (e.g. confidence intervals)
☒	☐	For null hypothesis testing, the test statistic (e.g. *F*, *t*, *r*) with confidence intervals, effect sizes, degrees of freedom and *P* value noted<br>*Give P values as exact values whenever suitable.*
☒	☐	For Bayesian analysis, information on the choice of priors and Markov chain Monte Carlo settings
☒	☐	For hierarchical and complex designs, identification of the appropriate level for tests and full reporting of outcomes
☒	☐	Estimates of effect sizes (e.g. Cohen's *d*, Pearson's *r*), indicating how they were calculated

Our web collection on statistics for biologists contains articles on many of the points above.

Software and code

Policy information about availability of computer code

Data collection	No software was used to collect these data. The data was provided by Cuebiq through the Data for Good program (www.cuebiq.com/about/data-for-good/)
Data analysis	Python2 was used for the analysis and models performed in this work. Codes employed in this work are publicly accessible on the Figshare repository at the link https://figshare.com/s/fe0a276da2cbca6d599d with DOI 10.6084/m9.figshare.19780192

For manuscripts utilizing custom algorithms or software that are central to the research but not yet described in published literature, software must be made available to editors and reviewers. We strongly encourage code deposition in a community repository (e.g. GitHub). See the Nature Portfolio guidelines for submitting code & software for further information.

Data

Policy information about availability of data

All manuscripts must include a data availability statement. This statement should provide the following information, where applicable:

- Accession codes, unique identifiers, or web links for publicly available datasets
- A description of any restrictions on data availability
- For clinical datasets or third party data, please ensure that the statement adheres to our policy

Researchers may request access to Cuebiq data by submitting proposals through Cuebiq’s “Spectus Social Impact” program (https://spectus.ai/social-impact/). Projects are considered on a case-by-case basis with priority given to projects that generate positive social impact and explore novel use cases and methodological

lines of inquiry. Projects are subject to review by Cuebiq's Privacy Council, and may require additional review by Researcher institutions.
Heathrow airport boundaries are available at https://www.openstreetmap.org/way/185882029#map=14/51.4693/-0.4537 under the license Open Data Commons Open Database Licence (ODbL).

Human research participants

Policy information about studies involving human research participants and Sex and Gender in Research.

Reporting on sex and gender	This information has not been collected.
Population characteristics	See above
Recruitment	No recruitment was carried out since the study is based on GPS mobility data from smartphone application records collected by Cuebiq on anonymized individuals who have opted-in to the service through a GDPR compliant framework. In order to preserve the privacy of individuals living in the neighboring areas, we exclude from the analysis movements outside the airport perimeter.
Ethics oversight	No ethic oversight was needed for this study since the work uses a synthetic population (not a real one) obtained by aggregating fully anonymized data records collected by Cuebiq on individuals who have opted-in to the service through a GDPR compliant framework.

Note that full information on the approval of the study protocol must also be provided in the manuscript.

Field-specific reporting

Please select the one below that is the best fit for your research. If you are not sure, read the appropriate sections before making your selection.

☐ Life sciences ☒ Behavioural & social sciences ☐ Ecological, evolutionary & environmental sciences

For a reference copy of the document with all sections, see nature.com/documents/nr-reporting-summary-flat.pdf

Behavioural & social sciences study design

All studies must disclose on these points even when the disclosure is negative.

Study description	Our work is a quantitative-methods study that involves aggregated GPS mobility data from smartphone application records collected by Cuebiq on anonymized individuals who have opted-in to the service through a GDPR compliant framework. The purpose is to build a temporal contact network of the airport area and perform epidemic modeling in order to understand how to counteract disease spreading within such environment and avoid exportation of infections to multiple countries through an optimized spatial immunization strategy.
Research sample	The sample used in the analyses accounts for 206,043 agents whose trajectories were recorded from February to August 2017. The research sample is representative of individuals' trajectories inside Heathrow airport since it is close in numbers to the individuals present on average at Heathrow daily as recorded by official sources https://www.heathrow.com/company/about-heathrow/performance/airport-operations/traffic-statistics. No information on age, sex and gender has been collected.
Sampling strategy	No statistical methods to predetermine the sample size was applied. Sample size was obtained from aggregating all the dataset trajectories from February to August 2017. The sample used in the analyses was chosen to recover the average number of individuals present at Heathrow daily as recorded by official sources https://www.heathrow.com/company/about-heathrow/performance/airport-operations/traffic-statistics.
Data collection	Cuebiq collects first-party data from anonymized smartphone users who provided informed consent to data collection for research purposes. As a GDPR compliant datasource, Cuebiq facilitates opt-out requests from users, while also completing requests for portability (providing a user's data to them on request) and erasure. All research projects undergo review and require approval by Cuebiq's Privacy Council.
Timing	The data includes individuals' trajectories inside the airport from February to August 2017.
Data exclusions	In order to preserve the privacy of individuals living in the neighboring areas, we exclude from the analysis movements outside the airport perimeter.
Non-participation	This does not apply since recruitment was not carried out and this is not an "in-person" experiment.
Randomization	Randomization was not applied since this is not an experiment with individuals in person, but involves modeling of epidemic spreading on collected mobility data of anonymized individuals trajectories, hence individuals do not need to be assigned to any group.

Reporting for specific materials, systems and methods

We require information from authors about some types of materials, experimental systems and methods used in many studies. Here, indicate whether each material, system or method listed is relevant to your study. If you are not sure if a list item applies to your research, read the appropriate section before selecting a response.

Materials & experimental systems

n/a	Involved in the study
☒	☐ Antibodies
☒	☐ Eukaryotic cell lines
☒	☐ Palaeontology and archaeology
☒	☐ Animals and other organisms
☒	☐ Clinical data
☒	☐ Dual use research of concern

Methods

n/a	Involved in the study
☒	☐ ChIP-seq
☒	☐ Flow cytometry
☒	☐ MRI-based neuroimaging
